# Cross-Linking Agents for Electrospinning-Based Bone Tissue Engineering

**DOI:** 10.3390/ijms23105444

**Published:** 2022-05-13

**Authors:** Dong-Jin Lim

**Affiliations:** Department of Otolaryngology Head & Neck Surgery, University of Alabama at Birmingham, Birmingham, AL 35294-0012, USA; daniel.djlim@gmail.com

**Keywords:** electrospinning, cross-linking agents, glutaraldehyde, 1-ethyl-3-(3-dimethylaminopropyl)-carbodiimide, genipin, citric acid, bone tissue engineering

## Abstract

Electrospun nanofibers are promising bone tissue scaffolds that support bone healing due to the body’s structural similarity to the extracellular matrix (ECM). However, the insufficient mechanical properties often limit their potential in bone tissue regeneration. Cross-linking agents that chemically interconnect as-spun electrospun nanofibers are a simple but effective strategy for improving electrospun nanofibers’ mechanical, biological, and degradation properties. To improve the mechanical characteristic of the nanofibrous bone scaffolds, two of the most common types of cross-linking agents are used to chemically crosslink electrospun nanofibers: synthetic and natural. Glutaraldehyde (GTA) is a typical synthetic agent for electrospun nanofibers, while genipin (GP) is a natural cross-linking agent isolated from gardenia fruit extracts. GP has gradually gained attention since GP has superior biocompatibility to synthetic ones. In recent studies, much more progress has been made in utilizing crosslinking strategies, including citric acid (CA), a natural cross-linking agent. This review summarizes both cross-linking agents commonly used to improve electrospun-based scaffolds in bone tissue engineering, explains recent progress, and attempts to expand the potential of this straightforward method for electrospinning-based bone tissue engineering.

## 1. Introduction

Bone tissue engineering (BTE) is a multidisciplinary field of engineering and life science that develops improved technologies that maximize clinical capabilities in treating skeletal defects [1]. Instead of transplanting donated bone grafts, BTE is composed of a synthetic extracellular matrix (ECM) called a scaffold, osteoblasts and related cells, and growth factors that regulate bone formation. BTE can overcome bone grafts’ common drawbacks, including donor insufficiency, supply limitations, and immune rejection [2]. The technologies include scaffolds that temporarily perform the extracellular matrix (ECM) function for the developing bone tissue [3]. Since the inception of the first generation of biomaterials in the 1960s, synthetic polymers (e.g., polymethyl methacrylate [PMMA] and polyetheretherketone [PEEK]), metals (e.g., titanium or titanium alloys), and ceramics (e.g., alumina and zirconia) are materials usually used in the fabrication of common bone scaffolds [4]. However, recent findings on the natural process of bone regeneration move the selection of scaffolds towards more fibrous or porous biocompatible materials due to their close resemblance to the native bone ECM [5]. By recapitulating essential features in native bone ECM, fibrous or porous scaffolds can facilitate the initial attachment, the growth, and the terminal differentiation of bone. Utilizing electrostatic forces, electrospinning can create multiple bundles of nano- or micron-size fibers such as native bone ECM with different biomaterials suitable for BTE [6,7]. The versatility and ease of continuous production of nanofibers with nano and micron size diameters from a variety of materials using electrospinning enable us to design better artificial ECM scaffolds with high porosity and interconnection, which are requirements for BTE scaffolds [7]. Nevertheless, the inadequate mechanical strength of ECM scaffolds in load-bearing BTE applications is one of the challenges involved in this simple and economical production method. As a result, numerous approaches, including a crosslinking technique, have been employed in BTE scaffolds design [8]. Crosslinking is a chemical modification approach commonly used for ensuring mechanical and chemical stability of nanofibrous scaffolds. It is the process of covalent bond formation between two or more molecules, mostly in polymers. An ideal crosslinking agent should help enhance the mechanical stability of fibrous scaffolds without cytotoxic effects. In addition to ensuring mechanical and chemical stability, crosslinking improves cell–matrix interactions, fibrous morphology retention, and biocompatibility by altering the antigenic sites of materials that induce the antigenicity [9,10]. Recent advancements have been made in designing and manufacturing scaffolds for BTE applications, particularly in utilizing crosslinking strategies for BTE. Even if several studies have addressed the importance of cross-linking agents for improving electrospun nanofibers for tissue engineering and BTE, this study has some unique highlights: (1) recently updated examples of using cross-linking agents have been addressed; (2) citric acid, which had been less used for crosslinking electrospun scaffolds, has been comprehensively reviewed; and (3) based on this review, a recent growing trend has been summarized.

## 2. Cross-Linking Agents

**Figure 1:**
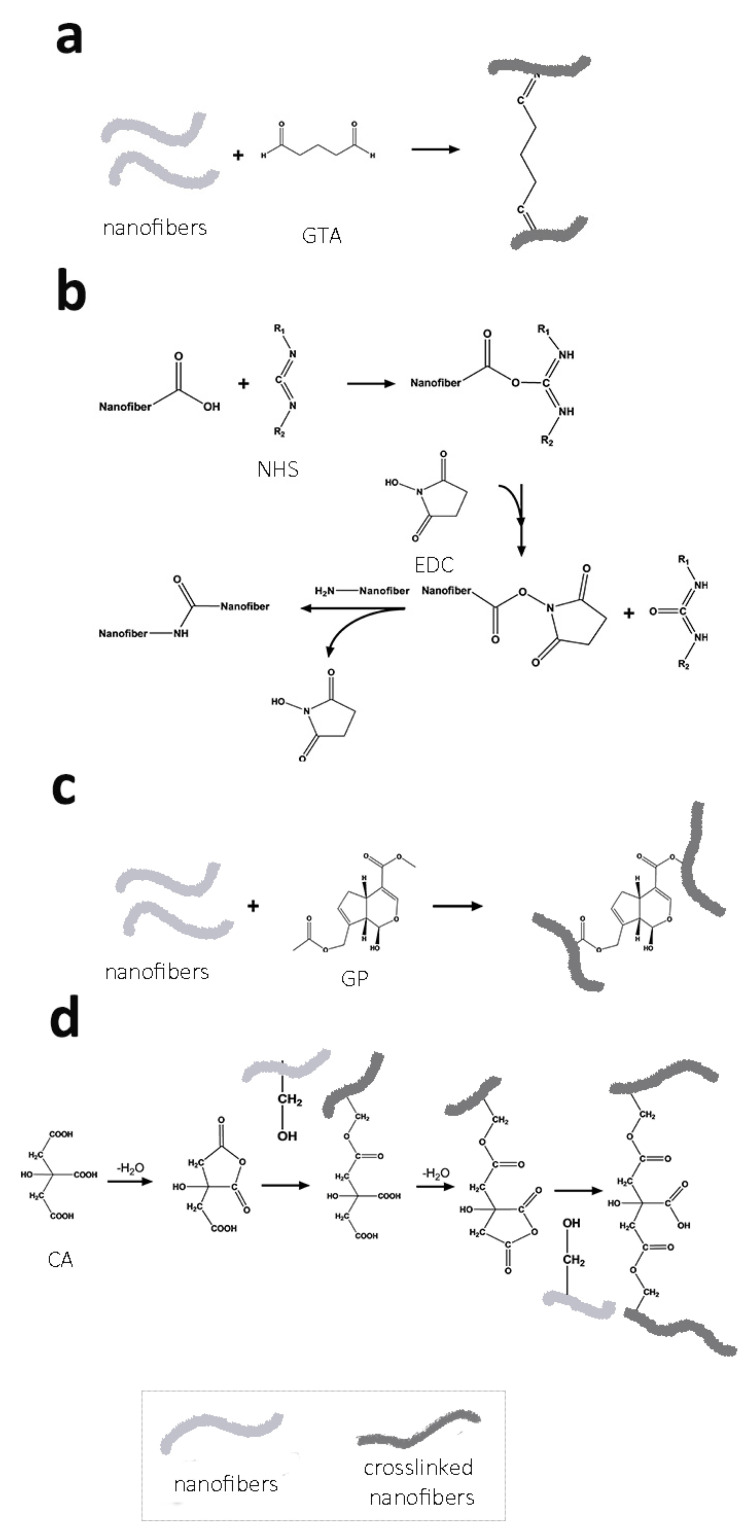
The illustrative mechanisms of cross-linking agents. (**a**) glutaraldehyde (GTA), (**b**) 1-ethyl-3-(3-dimethylaminopropyl)-carbodiimide (EDC), (**c**) genipin (GP), and (**d**) citric acid (CA).

To create a suitable scaffold for BTE, a variety of crosslinking strategies have been studied. Crosslinking electrospun-based bone scaffolds are based on connecting the functional groups exposed from electrospun nanofibers. Cross-linking agents connect polymer chains by chemically bonding more than two reactive ends. Since all electrospun-based bone scaffolds should be placed in the body, they must have excellent physical stability. The cross-linking strategies can maintain the nanofibrous features of electrospun scaffolds similar to native bone ECMs. Further cross-linking treatments can prevent the disintegration of nanofibers while offering improved mechanical properties. The mechanically improved electrospun scaffolds can withstand numerous biophysical stresses such as pH, enzymes, and physical loading within the body. There are three main categories of cross-linking treatments: (1) physical methods, (2) enzymatical methods, and (3) chemical methods. The physical methods are subdivided into dehydrothermal treatment (DHT) and exposure to γ-radiation, ultraviolet (UV), and plasma. For example, as-spun collagen nanofibers were dehydrothermally crosslinked at high temperatures (e.g., 140 °C for 1 day) [11]. However, only DHT treatment was inferior to a combinational cross-linking treatment with a chemical cross-linking agent, 1-ethyl-3-(3-dimethyl aminopropyl)-carbodiimide (EDC) or EDC alone. Under exposure to γ-radiation, trially isocyanurate-containing poly(L-lactide) (PLLA) electrospun nanofibers have been crosslinked [12]. Modified collagen electrospun nanofibers were photochemically crosslinked under UV [13]. Low-pressure air plasma was used to crosslink allylamine-modified polysuccinimde nanofibers [14]. Likewise, a gelatin nanofiber was crosslinked by non-equilibrium atmospheric pressure plasma generated by an atmospheric dielectric barrier discharge (DBD) plasma [15]. For enzymatical methods, microbial transglutaminase (mTG) has been used since mTG is the widely adopted safe enzyme for improving the texture of final products and product stability [16]. This review highlights chemical cross-linking strategies as the typical approach to crosslinking electrospun scaffolds (Figure 1). The most employed cross-linking agents are synthetic or natural [9]. Among the various synthetic chemical crosslinkers, glutaraldehyde (GTA) is one of the most commonly used synthetic agents, while a natural agent named genipin (GP), extracted from Gardenia fruits, has also gathered popularity [17].

### 2.1. Synthetic Cross-Linking Agents

#### 2.1.1. Glutaraldehyde

**Figure 2:**
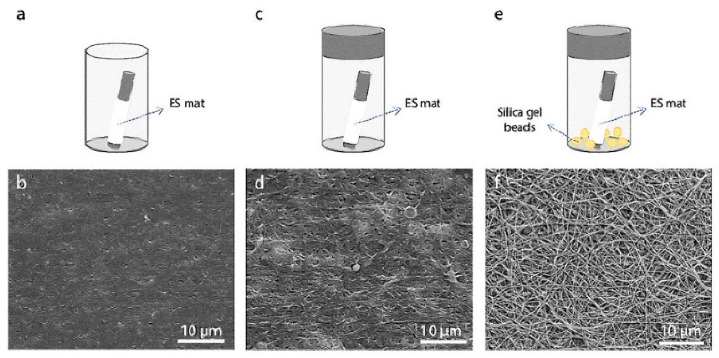
Effect of different evaporation conditions on fiber morphology after the crosslinking process (acetonitrile, 50 mM EDC, 8 h). SEM micrographs of gelatin nanofibers: (**a**,**b**) air drying, (**c**,**d**) evaporation in a closed vessel, and (**e**,**f**) evaporation in a closed container containing silica gel beads. Scale bars represent 10 µm. Reproduced from [30] and is licensed under a Creative Commons Attribution 4.0 International License.

Glutaraldehyde (GTA) is a bifunctional agent with highly reactive aldehyde groups capable of bonding with amines, thiols, phenols, hydroxyl, and imidazole groups [18]. Due to the strong affinity for amines, GTA is a general cross-linking agent for proteins, where the amine groups of lysine or hydroxylysine in proteins chemically react with the aldehyde group of GTA [19]. The GTA-mediated crosslinking of amines often occurs under neutral to basic conditions. GTA is much more efficient than other aldehydes since it gives thermally and chemically stable crosslinked forms [20]. The use of GTA has been widespread since GTA is cheap and efficient in stabilizing scaffolds [21]. Due to the resistance of GTA-crosslinked tissues against biological degradation, GTA has been used to prepare bioprostheses (e.g., heart valves, vascular grafts, and elastic cartilages). For example, GTA-mediated bioprosthetic heart valves (BHVs) can be prepared from porcine tissues [22,23]. However, GTA-treated tissues often lead to unwanted calcification, limiting the long-term durability of bioprostheses [24]. Additional treatment with amino acids and citric acid (CA) has been reported to reduce the cytotoxicity of GTA [25,26]. The involvement of phospholipids, cholesterols, and circulated free calcium post-implantation is thought to induce calcification and remove phospholipids and cholesterols from donor’s tissue by reagents such as ethanol and ethanol-based solutions, which have been studied to reduce bioprostheses calcification [27,28]. Besides, GTA-treated electrospun scaffolds showed good biocompatibility without significant in-vitro cytotoxicity [29]. The amount of GTA, scaffolds to be fixed, and treatment conditions would contribute to the final quality of GTA-treated electrospun scaffolds. For example, a study indicated that a controlled environment in a GTA vapor resulted in excellent morphological integrity (Figure 2) [30]. This study placed silica gel beads in the GTA-vaporized atmosphere to reduce humidity. 

In-situ crosslinking with GTA (0.5 M) made PVA electrospun nanofibers tough. Approximate six-fold increases in the tensile strength and significant elongation characteristics are observed [31]. The GTA crosslinking also changes the surface property of the electrospun scaffold. Because of the reduction of hydrophilic groups of gelatin nanofibers after treatment, GTA-cured gelatin nanofibers had better hydrolytic resistance against water than untreated gelatin nanofibers [32]. 

#### 2.1.2. 1-Ethyl-3-(3-dimethylaminopropyl)-carbodiimide

1-ethyl-3-(3-dimethylaminopropyl)-carbodiimide (EDC) is another common crosslinker for electrospun-based scaffolds. It is known as a zero-length crosslinker that chemically activates a molecule to covalently bond to another molecule. EDC has been used to create cross-linked scaffolds from collagen, gelatin, fibrinogen, and biopolymers [33,34,35,36,37,38]. In terms of EDC crosslinking chemistry, EDC is a water-soluble carbodiimide that activates the side groups of proteins to form a stable bond with other side groups and to form the ester bonds between the hydroxyl and carboxyl group of biopolymers [39]. EDC facilitates the formation of active O-acylisourea derivatives between the EDC and the carboxyl group of given scaffolds, followed by nucleophilic substitution with a strong nucleophile (e.g., primary amine). It becomes non-toxic N-substituted urea when EDC is not involved in the bond formations [40]. Instead of using direct EDC, a combination of EDC with either N-hydroxysuccinimide (NHS) or the more soluble sulfo-NHS (N-hydroxysulfosuccinimide) has been utilized to increase the coupling efficiency by a carboxyl group under the EDC reaction, which forms a stable NHS ester compared to the O-acylisourea [41]. For example, to create a gelatin-crosslinked electrospun scaffold, an in-situ EDC/NHS crosslinking strategy was used to create a biocompatible crosslinked gelatin electrospun [42]. 

The EDC treatment can also improve mechanical properties, similar to GTA crosslinking electrospun scaffolds. A study showed that the EDC-treated collagen II nanofibers were crosslinked successfully and that the EDC treatments (200 or 20 mM), irrespective of the presence of NHS, exhibited enhanced mechanical properties [43]. The EDC/Sulfo-NHS-treated gelatin nanofibers exhibited an eight-fold increase in the tensile strength, showing 16.06 ± 1.76 MPa and 2.00 ± 0.32 MPa for the untreated gelatin nanofibers [44]. For the elastic modulus, each value was reported as 1.813 ± 0.385 GPa and 0.108 ± 0.027 GPa, respectively.

### 2.2. Natural Cross-Linking Agents

#### 2.2.1. Genipin

**Figure 3:**
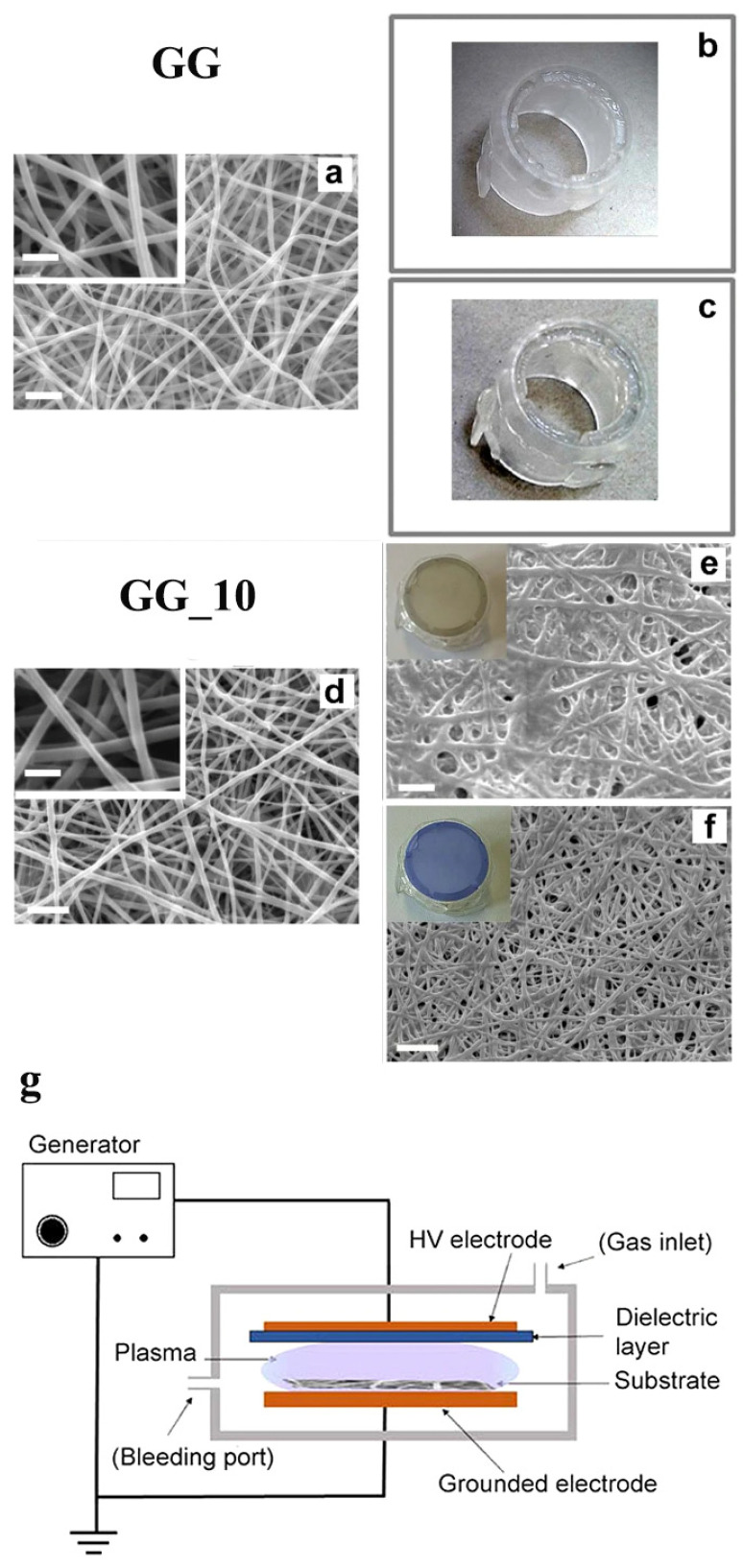
Scanning electron microscope (SEM) images and pictures of genipin-containing gelatin (GG) electrospun nanofibrous mats with 10 min plasma treatments (GG_10). Both GG and GG_10 mats were placed in double distilled water (DDW) or phosphate buffer (PB, pH 7.4). (**a**) as-spun GG, (**b**) GG_DDW, (**c**) GG_PB, (**d**) GG_10, (**e**) GG_10 DDW, (**f**) GG_10 PB, and (**g**) a schematic image of an atmospheric dielectric barrier discharge (DBD) plasma. Scale bars: (**a**,**d**,**e**) = 2 μm; (**f**) = 5 μm. Inset: higher magnification of mats GG and GG_10. Scale bar = 1 μm. Reproduced from [15] and is licensed under a Creative Commons Attribution 4.0 International License.

Genipin (GP) is a natural cross-linking agent with low toxicity obtained from the gardenia fruit. It is founded on one of its parent compounds, geniposides, in the fruits of *Gardenia Jasminoides* Ellis [45]. GP quickly becomes a blue pigment when spontaneously reacted with amino acids (e.g., glycine, leucine, and glutamic acid) [46]. According to multiple mechanistic studies, GP can only react with primary amines [47,48,49]. In natural conditions, GP in the fruits produces a brilliant blue pigment, a food colorant known as gardenia blue [50]. GP has been reported as a naturally occurring gentile cross-linking agent compared to GTA. In-vitro cellular cytotoxicity tests using 3T3 fibroblasts (BALB/3T3 C1A31-1-1) demonstrated that GP is 1000 times less cytotoxic than GA [51]. Moreover, GP showed good in-vivo biocompatibility, where the authors proved that GP-crosslinked chitosan microspheres were superior to GA-crosslinked microspheres [52]. While GA-crosslinked microspheres still evoked inflammatory reactions after a 12-week implantation in the skeletal muscle of Wistar rats, no apparent inflammatory cells were found in the group of GP-crosslinked microspheres. Numerous studies have been documented using GP to create functional biomaterials based on chitosan, one of the widely used biomaterials featuring excellent biocompatibility, nontoxicity, and flexible chemical tunability [53,54]. In addition, GP has been utilized for creating tissue-derived ECM scaffolds, including cardiac matrix hydrogel, decellularized nucleus pulposus hydrogel, and spinal cord ECM hydrogel [55,56,57]. As one of the major ECMs in the body, collagen has often been subject to further crosslinking with GP to create a functional scaffold. A GP crosslinked collagen hydrogel was used for creating a hybrid scaffold containing bone-marrow-derived stem cells (BMSCs) and cadmium selenide (CdSe) quantum dots (QDs), which produce reactive oxygen species (ROS) that induce chondrogenic differentiation [58]. Due to GP, the scaffold exhibited enough stiffness to promote chondrogenesis while releasing ROS within the hybrid hydrogel when triggered by a 595 nm light. For intervertebral disc degeneration, a GP crosslinked type II collagen scaffold was successfully fabricated and used for differentiating adipose-derived stem cells (ADSCs) into nucleus pulposus for the disc regenerative therapy [59]. Gelatin is another well-known biomaterial for the GP crosslinking strategy. A study using a collagen–gelatin–genipin(GP)–hydroxyapatite composite containing human primary osteoblasts demonstrated that the composite is suitable for bone tissue engineering [60]. Similarly, a study used GP to create a bone-inducing composite composed of collagen, hydroxyapatite, and alendronate (ALN) [61]. Hydroxyapatite (Ca_10_(PO_4_)_6_(OH)_2_, HA) is an inorganic component of bone, while ALN is a drug for osteoporosis [62,63]. Interestingly, GP treatment can be utilized in a combinational crosslinking strategy. A study demonstrated that GP-containing gelatin electrospun nanofibers were crosslinked faster when treated together with non-equilibrium atmospheric pressure plasma [15]. By reducing the contact of the aqueous solution during the combinational crosslinking treatment, the authors obtained completely crosslinked gelatin electrospun fibrous mats with improved mechanical and morphological features. Even after placing in either double distilled water (DDW) or phosphate buffer (PB, pH 7.4, and ionic Strength = 0.26 M), the GP-containing gelatin nanofibers (GG) treated with an additional 10 min of plasma showed intact integrity. In contrast, no plasma treatment showed the completed dissolution of GG samples either in DDW or PB (Figure 3). In a study using gelatin (G) and genipin-containing gelatin (GG) electrospun nanofibers, the incorporation of genipin significantly affected the mechanical properties of the final electrospun scaffolds [64]. Based on results, 15 ± 5% of the GG electrospun nanofibers were crosslinked, while a further crosslinking treatment in 5% GP for 7 days made GG electrospun nanofibers much more crosslinked (92 ± 5%). Significant changes in parameters regarding the mechanical properties were reported. Compared to G samples, Young’s modulus was increased by four-fold; the stress at break was increased by 3.5-fold; and the deformation at break was reduced by 10-fold. 

#### 2.2.2. Citric Acid

Citric acid (CA) is a natural organic tricarboxylic acid found in citrus fruits [65]. Two typical citrus fruits, lemons and limes, have been reported to contain nearly 8% of CA in dry weight [66]. CA is known as a flavoring, a sequestering, or a buffering agent and is used as the main ingredient in a variety of industrial sectors [67]. Interestingly, CA has also been studied as a versatile monomer to create biocompatible elastomeric materials. Poly(diol citrate) was synthesized and evaluated. Based on the three functional groups of CA (three carboxyl groups and one hydroxyl group), poly(1,8-octanediol-co-citric acid) (POC) was successfully synthesized in mild and straightforward conditions and studied to demonstrate its potential in tissue engineering [68]. CA is a promising mild cross-linking agent for developing several materials in multiple sectors, including the food industry, pharmaceutical industry, cosmetics, tissue engineering, and environmental science (Table 1). CA can increase the performance of biocompatible and edible films. For food applications, a study compared physical characteristics between non-crosslinked starch films and CA-crosslinked starch films and found that CA crosslinking resulted in an approximately 1.5-fold increase in the strength of starch films compared to non-crosslinked starch films [69]. Starch has multiple hydroxy groups, three hydroxy groups in each anhydroglucose monomer and the hydroxyl group belonging to the reduced end of each starch. CA and starch can undergo esterification reactions, producing CA mono-, di-, and tri-esters [70]. CA-cured aga/fish gelatin films have been fabricated at high temperatures (90 °C and 105 °C) [71]. Starch films are crosslinked with CA to extend the use of starch materials in food applications.

CA crosslinking helps tune starch’s hygroscopic and hydrophilic characteristics in food packaging materials, leading to the improved barrier property against water [69]. A study proved that CA crosslinking strategy could improve the moisture-resistance of poly(vinyl alcohol) (PVOH), which is suitable for food packings due to its ultra-low gas permeability [78]. Moreover, the authors conferred antimicrobial properties of CA-cured PVOH film by incorporating grapefruit seed extract (GSE), showing broad anti-microbial spectrums [79,80]. Utilizing the crosslinking property of CA, two biocompatible polymers, a natural carboxymethylcellulose (CMC) and poly(ethylene oxide) (PEO), were crosslinked into a composite hydrogel capable of methylene blue (MB), which is a good model drug with different pharmaceutical benefits for medical applications [81,82]. Similarly, carboxymethyl sago starch (CMSS) was cross-linked with CA to form a hydrogel potentially useful for drug delivery applications [83]. Xanthan gum produced from fermented sugar cane with *Xanthomonas campestris*, a well-known thickener in cosmetic applications, was esterified with CA at high-temperature incubation (165 °C for 7 min) to create a xanthan–citric acid hydrogel (XCA hydrogel) [84]. CA was served as a cross-linking agent in the case of a mixture of starch and xanthan gum [85]. The authors successfully fabricated a starch/xanthan gum hydrogel through extrusion and thermos pressing processes. A significant curing reaction was made with sodium hypophosphite (SHP), which acted as a catalyst in the reaction.

**Figure 4:**
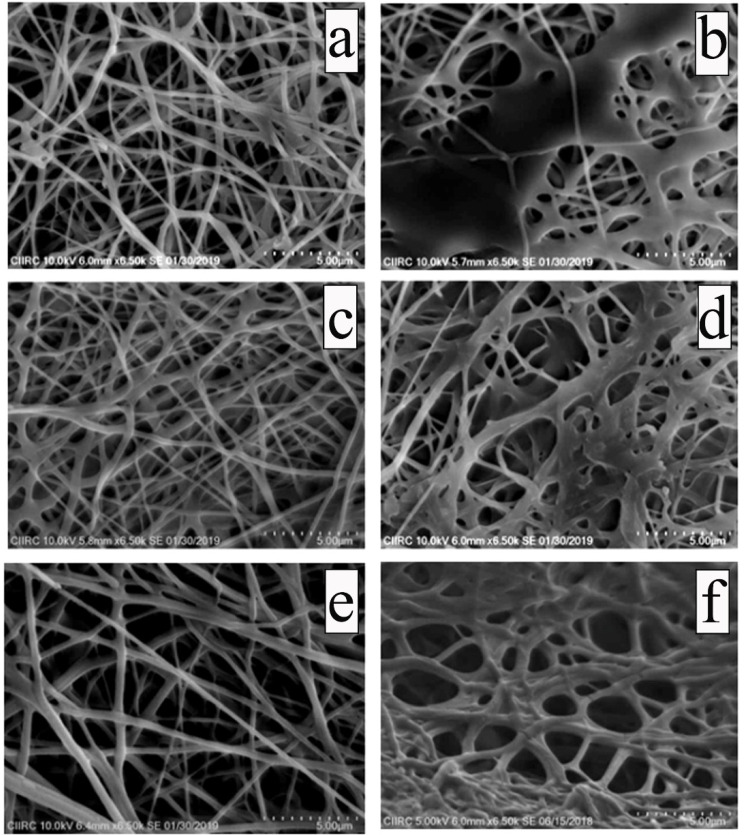
Scanning electron microscope (SEM) images of 5% (**a**,**b**), 10% (**c**,**d**), and 15% (**e**,**f**). Citric acid (CA) crosslinked electrospun nanofibers before (**a**,**c**,**e**) and after (**b**,**d**,**f**) immersion in water. Reproduced from [86] with permission, copyright Elsevier, 2020.

For medical applications, CA has also been evaluated to increase the potential of biocompatible polymers. For example, a well-used synthetic polymer, poly (vinyl) alcohol, was employed to form PVA electrospun followed by a CA treatment, leading to crosslinked PVA nanofibers, which were stable in water after a 72 h-incubation (Figure 4) [86]. Moreover, the crosslinked PVA nanofibers were well-served as a substrate for the attachment and proliferation of NIH 3T3 mouse fibroblast cells. Two carboxyl groups in a CA molecule were utilized to improve the osteogenic differentiation of human mesenchymal stem cells (hMSCs) within a cellulose-based hydrogel via a partial crosslinking strategy [87]. The idea of this study was that the introduction of hydrophilic carboxylic groups via a CA crosslinking facilitates the surface wettability of cellulose derivatives, sodium salt of carboxymethyl cellulose (CMCNa), and hydroxyl ethyl cellulose (HEC), thereby promoting hMSC cellular responses towards bone regeneration. Cell sheet engineering (CSE) is another emerging tissue engineering field where CA crosslinking chemistry creates a synthetic and durable scaffold. For delivering native ECM and intact cellular components onto host tissue, methylcellulose (MC) hydrogel has widely been utilized for CSE. Still, MC hydrogel quickly becomes weaker in an aqueous environment, reducing the performance of MC. Otherwise, CA crosslinked MC hydrogel exhibited improved mechanical properties without cytotoxicity due to CA [88]. 

Modifying materials via CA-mediated crosslinking provides innovative materials for renewable and environmental technologies. Starch-glycerol-CA films were fabricated to show excellent biodegradability while reducing starch retrogradation, often limiting its performance in renewable material applications [89]. CA-cured CMC was also studied as a technology approach to removing environmental pollutants in water. The authors used a high concentration of CA, up to 20%, to form a CMC hydrogel with excellent adsorption efficiency and removal capacity. Based on this study, this CA-cured CMC hydrogel exhibited good absorption potential for pollutant removal [90]. 

In the abovementioned study, the tensile strength of the CA-curing PVA electrospun nanofibers was gradually increased with increasing CA concentrations [86]; 15%, 10%, and 5% CA crosslinked PVA nanofibers showed 7.6, 7.6, and 6.2 MPa, while 6.0 MPa for untreated PVA electrospun nanofibers. Inversely, the percentage of elongation of the CA-curing PVA electrospun nanofibers was 69, 55, and 85%, indicating that CA crosslinking creates a mechanically reinforced scaffold because untreated PVA was created electrospun nanofibers had 122%.

## 3. Cross-Linking Strategies for Bone Tissue Engineering

For recapitulating the native bone microenvironment for BTE, an ideal scaffold should have adequate mechanical properties supporting the regenerative potential of bone. As previously mentioned, cross-linking agents used for chemical crosslinking strategies quickly improve the electrospun’s performance, mimicking the native bone ECM [91]. With the aid of the efficient crosslinking potential of GTA vapor, fluoride, an inorganic ion capable of improving osteoblastic proliferation and collagen synthesis, was successfully incorporated into gelatin electrospun nanofibers [92]. This study used the GTA vapor technique for four days to complete the crosslinking reaction. To improve the mechanical strength of gelatin electrospun, a study incorporated hexagonal boron nitride sheets as an external filler before electrospinning and crosslinked them with a 1% GTA solution to create reinforced gelatin electrospun nanofibers [93]. GTA vapor made crosslinked Bombyx mori silk fibroin (SF)/gelatin nanofiber mats with different SF/gelatin blend ratios [94]. According to the author’s observations, a high gelatin ratio in the blending solution for electrospun results in much more efficient GTA crosslinking. Consequently, the final blended nanofibers demonstrated a better tensile strength than non-crosslinked mats. However, the potential toxicity of GTA-cured blended mats was also presented. EDC/NHS cross-linking treatment was utilized for creating crosslinked silk fibroin (SF)/gelatin type B (GB) electrospun scaffolds [95]. The authors can modulate the enzymatic degradability of the final SF/GB electrospun nanofibers by using EDC/NHS cross-linked SF/GB blended nanofibers obtained from different SF/GB blended solutions (e.g., as SF/GB ratios, 10/90, 20/80, 30/70, 40/60, and 50/50). To prevent the disintegration of collagen electrospun in aqueous crosslinking conditions, ethanolic EDC/NHS treatment was evaluated for crosslinking type II collagen fibrous electrospun matrices [43]. Based on the author’s observation, two different EDC concentrations (20 mM and 200 mM) in ethanol led to comparable crosslinking efficiency to the heated 50% GTA treatment. By an ethanolic EDC treatment for 18 h, a study made aligned and crosslinked PLGA/collagen electrospun nanofibrous matrices for bone tissue engineering [96]. A biomineralized scaffold is a promising approach in bone tissue engineering because deposited inorganic components confer the osteoconductive and osteoinductive properties to the scaffold. To create a biomineralized electrospun composed of collagen and oligosaccharide hyaluronic acid (oHA), which expects an angiogenic potential in vivo, 25% solutions of either GTA or EDC were placed for 2 days on the bottom of a desiccator together with as-spun fibrous sheets [97].

Chitosan electrospun nanofibers with hydroxyapatite (HA) were crosslinked with GP. When crosslinked with GP, HA containing crosslinked chitosan (CTS) electrospun composites demonstrated similar mechanical properties to the periosteum, which is a thin layer outside of bone composed of osteogenic and fibroblastic cells [98,99]. These CTS nanofibers were suggested for cranial and maxillofacial reconstruction [99]. Another study proved that GP crosslinked CTS electrospun nanofibers were more resistant to degradation than uncrosslinked CTS nanofibers without adverse cytotoxic effects [100]. In a similar approach, a hybrid electrospun made from a mixture of gelatin and poly(ε-caprolactone) (PCL) was crosslinked with GP, where as-spun nanofibers were immersed in 2% of a GP ethanolic solution for 24 h [101]. This study used a co-solvent solution mixed with 2,2,2-Trifluoroethanol (TFE) and acetic acid (0.2% *v*/*v*) and GP crosslinking to fabricate a uniform gelatin/PCL electrospun composite. Additionally, the authors proved the potential of prepared gelatin/PCL nanofibers in guided bone regeneration (GBR) membranes. The GP crosslinking strategy is also viable for more complex electrospun nanofibers. GP was used to create stable core-shell nanofibers, where icariin-loaded CTS microspheres were embedded inside of shell PCL/HA electrospun nanofibers [102]. Icariin (ICA) is a prenylated flavonol glycoside isolated from Epimedium herb, supporting bone formation [103]. Moreover, GP can create improved electrospun scaffolds fabricated by a co-electrospinning method that concomitantly generates interspersed electrospun from different polymers. Co-electrospun poly(l-lactic acid) (PLLA) and gelatin nanofibers were crosslinked with GP for cartilage–bone interfaces tissue engineering [104].

Another natural crosslinker useful for BTE is citric acid (CA), but CA is not a frequently used strategy. There are three possible explanations regarding the bottleneck that limits the popularity of CA in the electrospun-based BTE: (1) crosslinking reactions are usually more severe than those of other crosslinking agents, (2) most natural polymers actively cross-linkable with CA have not been common biomaterials (e.g., collagen and gelatin) applied in BTE, and (3) CA is a water-soluble agent. However, there is still enough room to use CA as a crosslinker for creating a novel electrospun-based scaffold in BTE. In situ CA-crosslinked alginate (AL)/polyvinyl alcohol (PVA) electrospun nanofibers were successfully fabricated, where 5% CA were blended with polymers before running electrospinning [105]. In this study, the post-two hours curing process at 140 °C resulted in CA-crosslinked electrospun nanofibers followed by a simulated body fluid (SBF) treatment suitable for BTE applications. Compared to non-crosslinked AL/PVA electrospun, the crosslinked AL/PVA maintained its integrity even after ten times immersion in water and drying cycle. The SBF treatment can confer a bone-forming ability on a nanofibrous electrospun scaffold. When treated with SBF, numerous biomaterials made from inorganic, organic, and metals can be integrated into natural bony structures [106]. Moreover, CA-crosslinked electrospun has the potential to deliver drugs for BTE. A study using CA containing a blend of chitosan and poly(ethylene oxide) (PEO) demonstrated that different heating treatments (150, 160, and 170 °C for 3 h) post-electrospinning formed crosslinked electrospun scaffolds and that the CA-crosslinked electrospun made in the highest heating treatment (170 °C) exhibited the extended-release of aspirin, a model drug, over time compared to other CA-crosslinked electrospun scaffolds [107]. Interestingly, a study used marine-sourced soluble collagen, denatured whole-chain collagen (DWCC), and dissolved them in a CA solution to prepare electrospinning solutions [108]. Then, different intra-fibrillar crosslinking conditions were evaluated with fabricated electrospun marine collagen fibers. Three electrospun nanofibers with other CA contents (the CA:DWCC molar ratios were 50:1, 125:1, and 260:1, respectively) were successfully fabricated. A heat treatment process (165 °C) was successfully fabricated and applied to the prepared electrospun DWCC nanofibers. Experiments found that the best intra-fibrillar crosslinked nanofibers CA-crosslinked were obtained when heat-treated at 165 °C on fabricated DWCC nanofibers from CA/DWCC blending solutions at a high molar ratio (CA:DWCC = 260:1, pH 3.5). Because collagen is one of the most relevant materials for BTE, such a crosslinking strategy would be useful for creating more advanced electrospun-based materials if biocompatibility is warranted. Moreover, a well-formulated blending solution containing both conventional water-insoluble collagen and CA has been used to evaluate the efficiency of different cross-linking agents on collagen-based electrospun nanofibers [109]. The authors used a benign ethanol/water solvent and a modified CA crosslinking strategy to create novel collagen-based electrospun nanofibers. Based on their observations, the CA-crosslinked collagen electrospun nanofibers kept their integrity intact for up to a month in phosphate-buffered saline at 37 °C. They promoted the attachment and growth of NIH 3T3 mouse fibroblasts, thereby proving the usability of CA as a natural cross-linking agent.

## 4. Conclusions and Future Directions

Electrospinning has been extensively studied in bone tissue engineering (BTE) to create a more natural bone extracellular matrix (ECM)-mimicking scaffolds. Electrospun-based bone scaffolds can easily possess biodegradability, osteoinductivity, osteoconductivity, and porosity but hardly exhibit the desired mechanical stability, an essential feature for BTE [7]. Several cross-linking agents have been widely studied to improve the mechanical property of as-spun nanofibers. In this review, the most commonly used cross-linking agents have been reviewed based on the origin of each agent. Both synthetic and natural cross-linking agents have continually been used in electrospun-based bone scaffolds. Interestingly, a growing new trend has adopted more natural agents in crosslinking electrospun-based bone scaffolds. In the case of synthetic crosslinking approaches, recent attempts can be summarized by (1) developing mild GTA protocols (e.g., vapor GTA), (2) using EDC chemistry, which produces non-toxic intermediates, and (3) exploring a combinational approach via using multiple cross-linking agents or adopting different cross-linking categories. Selecting and optimizing cross-linking protocols with proper agents are likely to be an essential step in creating suitable electrospun scaffolds in BTE. In addition, several anticipated directions of crosslinking strategies would be expected: (1) many more mild protocols of the chemical-based crosslinking strategy will be developed; (2) combinational use of both synthetic and natural cross-linking agents will frequently be attempted; (3) the other cross-linking treatments (i.e., physical methods and enzymatical methods) will be used together to develop a robust and mild method for modulating electrospun-based bone scaffolds; and (4) different natural cross-linking agents (e.g., proanthocyanidin [110] and epigallocatechin gallate [111]) should be explored to crosslink electrospun-based bone scaffolds as well in search of creating the best biomimetic scaffold for BTE. The further development of current protocols of chemical cross-linking agents or new cross-linking strategies would be an indispensable tool to increase the potential of the electrospun-based scaffold in BTE.

## Figures and Tables

**Table 1:** Examples of applications of citric acid (CA) ^1^.

Applications	Descriptions	Ref.
Pharmaceutically active substances, pharmaceuticals, personal care, and cosmetic products	Many active pharmaceutical ingredients (APIs) are supplied as their citrate salt. Citric acid is used to mask the bitter taste of drugs.	[67,72]
Food and beverage	Enhancing the activity of antioxidant preservatives (citrate is a powerful chelating agent for trace metal ions), Acidulant, and pH stabilizers.	[73]
Flavoring agent	Sharp and acid taste of citric acid can help mask pharmaceuticals’ unpleasant, medicinal taste.	[74]
Blood anticoagulant	Citrate chelates calcium, reducing the tendency for blood to clot.	[75]
Environmental remediation	Chelating agents sequestering heavy metals, including radioactive isotopes, and also easing the removal of hydrophobic organic compounds.	[76]

^1^ This table is a revisited table from an original table in [77], published and licensed under a Creative Commons Attribution 4.0 International License.

## Data Availability

Not applicable.
